# 735 Burns During the Pandemic: An Epidemiological Study of COVID-19's Impact on an Urban Burn Center

**DOI:** 10.1093/jbcr/irad045.210

**Published:** 2023-05-15

**Authors:** Deborah Choe, Paul Won, Joshua Abu-Ghazaleh, Zachary Collier, Justin Gillenwater, Haig Yenikomshian

**Affiliations:** Keck School of Medicine of USC, Los Angeles, California; University of Southern California, Pasadena, California; UC Berkeley, Los Angeles, California; Division of Plastic and Reconstructive Surgery, Keck School of Medicine of USC, Los Angeles, CA, Los Angeles, California; Keck Medicine of USC, Los Angeles, California; University of Southern California, Los Angeles, California; Keck School of Medicine of USC, Los Angeles, California; University of Southern California, Pasadena, California; UC Berkeley, Los Angeles, California; Division of Plastic and Reconstructive Surgery, Keck School of Medicine of USC, Los Angeles, CA, Los Angeles, California; Keck Medicine of USC, Los Angeles, California; University of Southern California, Los Angeles, California; Keck School of Medicine of USC, Los Angeles, California; University of Southern California, Pasadena, California; UC Berkeley, Los Angeles, California; Division of Plastic and Reconstructive Surgery, Keck School of Medicine of USC, Los Angeles, CA, Los Angeles, California; Keck Medicine of USC, Los Angeles, California; University of Southern California, Los Angeles, California; Keck School of Medicine of USC, Los Angeles, California; University of Southern California, Pasadena, California; UC Berkeley, Los Angeles, California; Division of Plastic and Reconstructive Surgery, Keck School of Medicine of USC, Los Angeles, CA, Los Angeles, California; Keck Medicine of USC, Los Angeles, California; University of Southern California, Los Angeles, California; Keck School of Medicine of USC, Los Angeles, California; University of Southern California, Pasadena, California; UC Berkeley, Los Angeles, California; Division of Plastic and Reconstructive Surgery, Keck School of Medicine of USC, Los Angeles, CA, Los Angeles, California; Keck Medicine of USC, Los Angeles, California; University of Southern California, Los Angeles, California; Keck School of Medicine of USC, Los Angeles, California; University of Southern California, Pasadena, California; UC Berkeley, Los Angeles, California; Division of Plastic and Reconstructive Surgery, Keck School of Medicine of USC, Los Angeles, CA, Los Angeles, California; Keck Medicine of USC, Los Angeles, California; University of Southern California, Los Angeles, California

## Abstract

**Introduction:**

Due to stay-at-home and social distancing orders driving the advent of virtual workplaces and classrooms, we hypothesized the COVID-19 pandemic altered the epidemiology of adult and pediatric burn injuries presenting to our large urban burn center.

**Methods:**

We retrospectively reviewed patients admitted to a metropolitan burn center during a 3-year period: one year prior to the COVID-19 pandemic (Control Year, 3/20/2019 - 3/19/2020) and the first 2 years of the pandemic (Year 1, 3/20/2020 - 3/19/2021; Year 2, 3/20/2021 - 3/19/2022). Data included burn etiology, severity, outcomes, and socioeconomic demographics.

**Results:**

There were 404 burn patients admitted during the pre-pandemic year (Control Year). Fewer patients were admitted during the pandemic years (Year 1 = 339; Year 2 = 374). The average age of burn patients was similar across the three years (Control Year 39.9 ± 22.6; Year 1 40.4 ± 22.1; Year 2 41.8 ± 21.9 years) with an increasing percentage of males (61%; 68%; 66%) and undomiciled individuals (15%; 19%; 23%) getting burned. Rates of scald, chemical, electrical, and blast burns were similar across years with greater variations in rates of flame (35%; 40%; 37%), contact (5.0%; 8.0%; 3.8%), and friction (0.0%; 0.6%; 1.3%) burns. Survival rates (95%; 94%; 94%) and hospital length-of-stay (11.4 ± 16.5; 11.6 ± 14.4; 12.4 ± 17.7 days) were similar across the 3 years with slightly longer but variable ICU stays during the pandemic (8.2 ± 13.6; 10.1 ± 15.8; 11.5 ± 17.7 days).

**Conclusions:**

Although the COVID-19 pandemic overwhelmed hospital systems and quarantine measures created altered home environments, our study suggests that burn severity and etiology were not significantly impacted by these changes. However, it does appear that the rate of burn injury decreased during the first year of pandemic lockdowns (Year 1) and started to increase towards pre-pandemic levels by Year 2 as lockdown restrictions were lifted. Flame, contact, and friction burns appeared to increase in frequency during Year 1 but normalized towards pre-pandemic levels by Year 2 as children returned to school. Analysis did show differences in ethnicity which may suggest the possibility of socioeconomic factors impacting who was getting burned during this time, but further studies are required to assess this potential disparity with greater nuance.

**Applicability of Research to Practice:**

This study improves our understanding of the ongoing impact of pandemic restrictions on burn injuries in cities and may help develop better prevention, education, and intervention strategies.

